# Case report: the first case of human infection by adult of *SPIROMETRA ERINACEIEUROPAEI* in VIETNAM

**DOI:** 10.1186/s12879-017-2786-x

**Published:** 2017-10-10

**Authors:** Anh Tran Le, Le-Quyen Thi Do, Huong-Binh Thi Nguyen, Hong-Ngoc Thi Nguyen, Anh Ngoc Do

**Affiliations:** 1 Department of Parasitology, Vietnam Military Medical University (VMMU), Phung Hung Street, Ha Dong Town, Hanoi, Vietnam; 2 Department of Infectious Disease, 103 Military Hospital, VMMU, Hanoi, Vietnam; 3 Department of Molecular Biology, National Institute of Malariology, Parasitology and Entomology (NIMPE), Luong The Vinh Street, Hanoi, Vietnam; 4 Department of Molecular Biology, National Institute of Malaria, Parasitology and Entomology (NIMPE), Hanoi, Vietnam; 5 Department of Parasitology, Vietnam Military Medical University (VMMU), Hanoi, Vietnam

**Keywords:** Case report, *Spirometraerinacei europaei*, Adult worm, Human, Vietnam, Molecular analysis

## Abstract

**Background:**

Tapeworms of the genus *Spirometra* include species whose larval stages can infect humans, causing a disease called sparganosis. Cases of human infection with adult worms are very rare and have been reported in Korea and China. Here we report the first case of human infection with an adult of *Spirometra erinaceieuropaei* in Vietnam.

**Case presentation:**

A 23-year-old male was admitted to 103 Military Hospital, Hanoi, Vietnam with fever, weight loss and epigastric discomfort. Preliminary diagnosis based on discovery of parasite eggs in his faeces incorrectly determined a fluke as the agent of the infection and praziquantel was prescribed. Two days later he passed out proglottids in his stool. The tapeworm was identified as *Spirometra erinaceieuropaei* using morphological and molecular tools.

**Conclusion:**

This is the first case of human infection with adult worm of *Spirometra erinaceieuropaei* in Vietnam.

## Background

*Spirometra* is a tapeworm genus in the order *Diphyllobothriidea* [1] that includes several species: *S. erinacei* (=*S. erinaceieuropaei*), *Sparganum mansoni*, *Spirometra mansonoides* and an aberrant form of *Spirometra proliferum* [2].

*Spirometra* has a complicated life cycle with three hosts. The definitive hosts are dogs, cats, and some other mammals where adult worms live in the small intestine and produce unembryonated eggs that are discharged in faeces. Once in fresh water, the unembryonated eggs hatch to become coracidia. The first intermediate hosts are copepods (Cyclops) taking up the coracidia, which develop into procercoid larvae in Cyclops. When fish, reptiles, or amphibians consume the copepods, they become the second intermediate host of *Spirometra*. Procercoid larvae penetrate the intestinal tract of the second intermediate host, become plerocercoid larvae (sparganum larvae), then migrate to the subcutaneous tissues and muscles. The cycle begins again as a definitive host takes up a second intermediate host. Many amphibians, reptiles and even mammals can become paratenic hosts when they are infected with spargana [3].

Humans can be accidental definitive, second intermediate or paratenic hosts as well. Larval stages of some species such as *S. erinaceieuropaei, S. mansoni, S. mansonoides, S. proliferum* can infect humans and cause a disease called sparganosis [4] which is endemic in Asian countries including China [5], Hong Kong [6], South Korea [7], Japan [8], and Thailand [9]. Cases of human infection with adult worms are very rare and have been reported in Korea and China [10, 11].

We report a case of human infection with adult of *S. erinaceieuropaei* identified by its morphology and genetic analysis in Vietnam. To our knowledge, this is the first case of adult *S. erinaceieuropaei* recovered from human in Vietnam.

## Case presentation

**Fig. 1:**
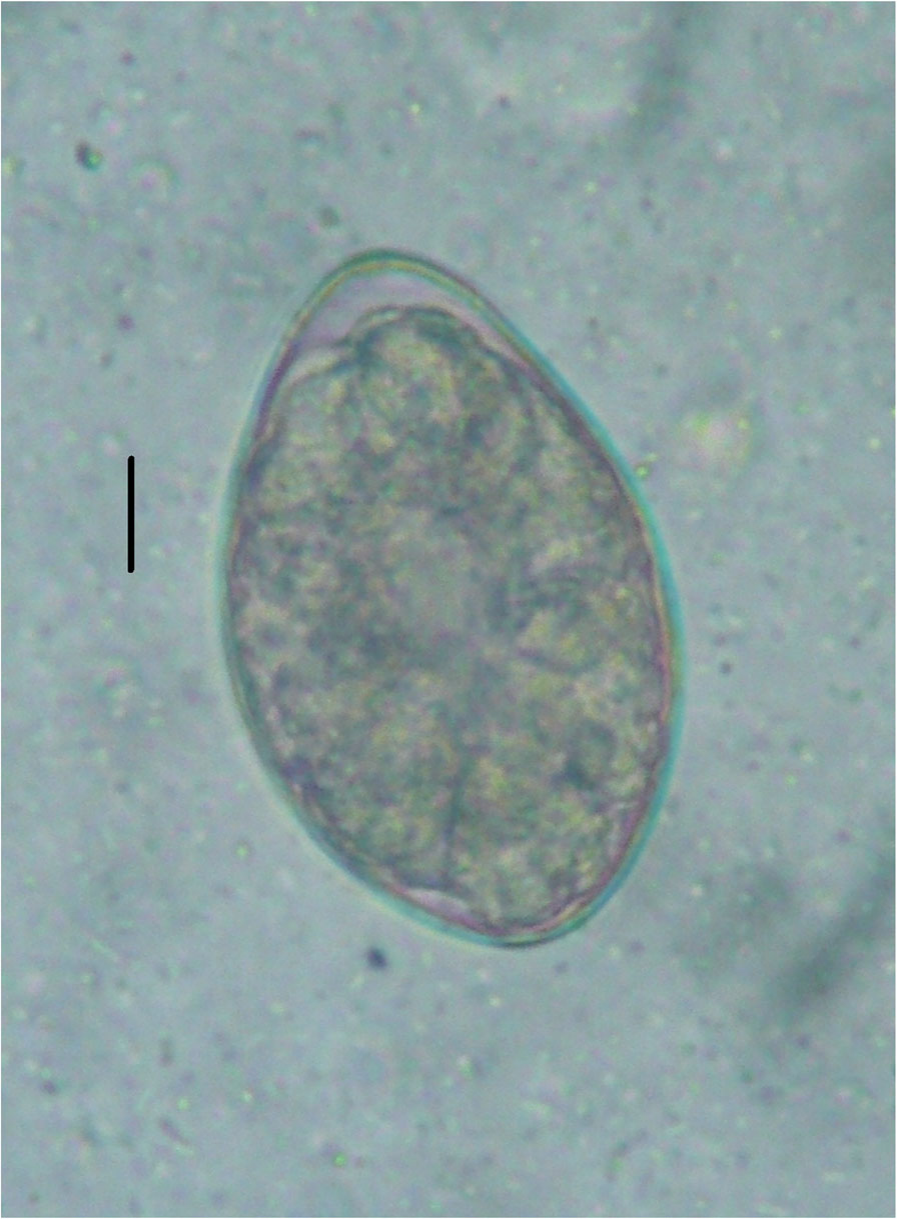
Ovoid, operculated eggs in stool (bar = 10 μm)

**Fig. 2:**
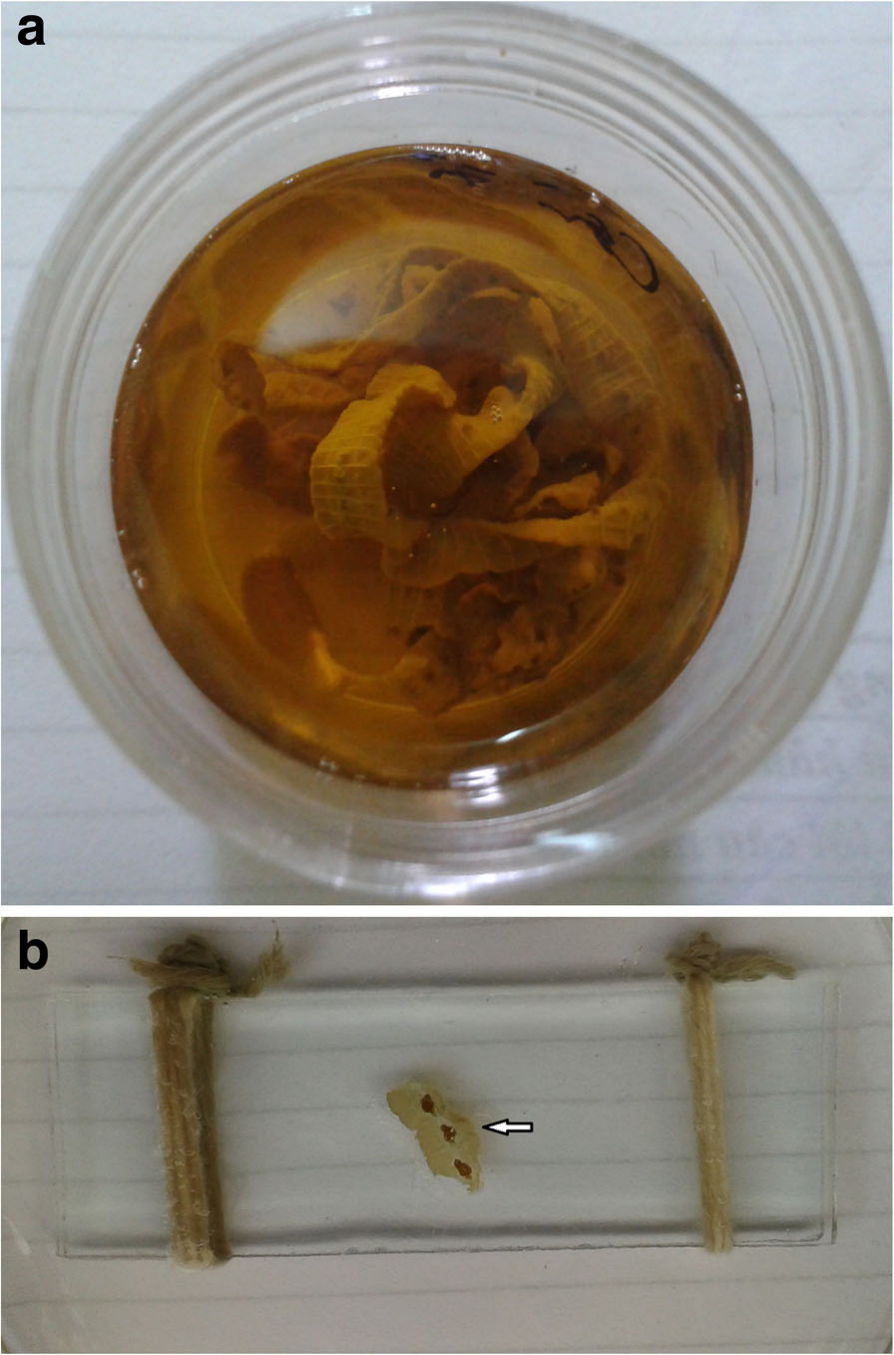
Wider than long proglottids in the stool after treatment with praziquantel (**a**: Poglottids in alcohol 70%. **b**: Poglottids on a slide (arrow)

A 23-year-old male was admitted to 103 Hospital (Ha Dong Town, Hanoi, Vietnam) on September 4th, 2012 with a sixteen-day history of fever and weight loss. The patient had slight epigastric pain migrating to right lower quadrant of the abdomen but no nausea or vomiting. In the first few days in hospital, his stool was watery but no mucus or blood was seen. Physical examination was normal. Complete blood count and liver function tests were performed and all were in a normal range. His blood was screened for hepatitis A, B and C, malaria, liver and lung flukes, *Toxocara* spp., *Strongyloides* spp. as well as cultured for microbacteria and the results were negative. Image studies (abdominal ultrasound and chest ray) were normal. With a sixteen-day history of fever the patient was diagnosed with sepsis and treated with a combination of two antibiotics (cefpirome and levofloxacin). Yet he showed no improvement in fever nor abdominal pain. On September 11th, a stool examination revealed a large quantity of ovoid-shaped eggs in sizes of about 60 × 40 μm (Fig. 1). Preliminary diagnosis of fluke was made and a single dose of praziquantel (1200 mg) was prescribed. On September 15th, he expelled pieces of tapeworm strobila, with off-white proglottids, wider than long (about 0.3 × 0.8 cm) in his stool (Fig. 2). Based on these morphological characteristics, the parasite was assigned to *Spirometra* sp. [12, 10]. Following the treatment of tapeworm, symptoms were quickly resolved. Stool tests for ova and parasite strobila two weeks later were negative. The patient fully recovered and was discharged some days later.

**Fig. 3:**
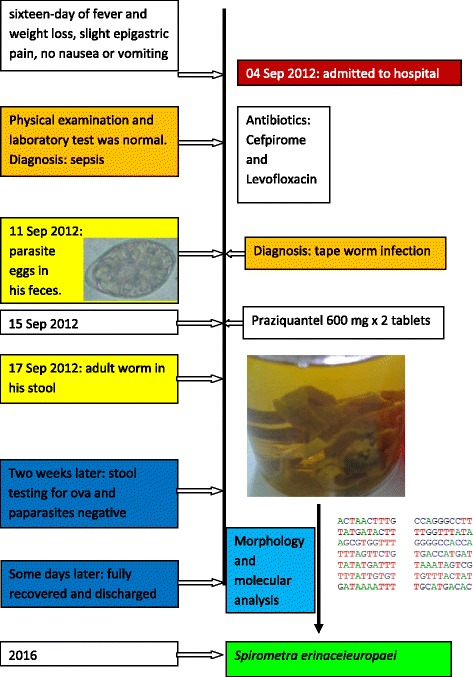
Timeline

To speciate the worm DNA was extracted from a snip of the worm strobila using QIAamp DNA Micro kit (QIAGEN) according to the manufacturer’s instructions. A PCR was performed following to protocol of Hyeong-Kyu Jeon [7]. The 440 bp product of PCR reaction with primers p1f and p1r was sequenced and analyzed by BLAST tool showing 99% identity with cytochrome c oxidase subunit 1 (Cox 1) gene of *S. erinaceieuropaei*. The sequence was submitted to GenBank (MF682495) Fig. 3.

## Discussion

The most common state of infection with this tapeworm is larvae. Only a few cases of human infection with adult worms have been reported so far [10, 11]. The patients usually suffered minor health problems and were successfully treated with praziquantel [13].

Vietnam and the south-eastern Asia region witness a large number of zoonotic diseases (taeniasis, trichinellosis) relating to the habit of eating raw meat [14]. Vietnamese people also traditionally practice using frog flesh as dressings for open wounds or eyes for medical purposes [15]. Water hygiene is another problem since clean water is not accessible in some areas. These factors lead to Vietnam being a conducive environment for diverse parasitological diseases in general and infections with *Spirometra* in particular. Although Vietnam is considered an endemic area of sparganosis [12], human clinical cases have been rarely reported, e.g. a subcutaneous sparganosis case published by Vortel el al. [16].

## Conclusion

This is the first case where an adult worm of *Spirometra erinaceieuropaei* was retrieved from human in Vietnam. The name of the tapeworm is *Spirometra erinaceieuropaei*, as confirmed by morphological and molecular tools. Further research to determine the exact epidemiological situation in Vietnam is necessary to facilitate appropriate preventive measures. Health education should be augmented to promote hygienic behaviors such as eating thoroughly cooked meat and drinking boiled water to prevent meat borne and waterborne diseases.

## Abbreviations

bp: Base pairs
Cox1: Cytochrome c oxidase subunit I
DNA: Deoxyribonucleic acid
PCR: Polymerase Chain Reaction
